# Association of cannabis use disorder with cardiovascular diseases: A two-sample Mendelian randomization study

**DOI:** 10.3389/fcvm.2022.966707

**Published:** 2022-10-06

**Authors:** Miao Chen, Yun-long Lu, Xiao-fan Chen, Zhen Wang, Liang Ma

**Affiliations:** ^1^Department of Cardiovascular Surgery, School of Medicine, The First Affiliated Hospital of Zhejiang University, Hangzhou, China; ^2^Department of Cardiology, School of Medicine, The First Affiliated Hospital of Zhejiang University, Hangzhou, China

**Keywords:** cannabis use disorder, cardiovascular diseases, Mendelian randomization study, GWAS - genome-wide association study, cardiovascular genetics

## Abstract

**Background:**

The use of cannabis has increased globally due to more regions decriminalizing marijuana use for therapeutic and recreational aims. Several observational studies have revealed that cannabis use is associated with an increased risk of adverse cardiovascular pathologies and diseases. Nevertheless, the causal associations between cannabis use and cardiovascular diseases remain unclear. Hence, we performed single-variable and multivariable Mendelian randomization (MR) to evaluate the association between cannabis use disorder and various cardiovascular diseases.

**Materials and methods:**

Summary statistics were collected from the largest-to-date genome-wide association studies (GWAS) of cannabis use disorder. The 12 SNPs for cannabis use disorder were used as instrumental variables in this study. MR estimates were pooled using a random-effects inverse-variance weighted (IVW) method. Simple median and weighted median methods were conducted as sensitivity analyses.

**Results:**

The genetic liability to cannabis use disorder was associated with an augmented risk of coronary artery disease, myocardial infarction, atrial fibrillation, heart failure, deep venous thrombosis, pulmonary embolism, and stroke. Except for stroke, the results were inconsistent in the sensitivity analyses. The overall patterns for the associations of cannabis use disorder with atrial fibrillation, heart failure, pulmonary embolism and stroke remained in multivariable MR analyses adjusting for potential mediators, including smoking, alcohol, body mass index, blood lipid, type 2 diabetes, hypertension, and depression. However, the association with coronary artery disease, myocardial infarction, and deep venous thrombosis did not persist in multivariable MR analyses. Mediation analysis demonstrated that smoking, body mass index, low-density lipoprotein, hypertension, and depression have more significant mediation effects, which suggests that these factors partly mediate the link from cannabis use disorder to coronary artery disease, myocardial infarction, and deep venous thrombosis.

**Conclusion:**

The genetic liability to cannabis use disorder was associated with a higher risk of atrial fibrillation, heart failure, pulmonary embolism, and stroke. The evidence for the association between cannabis use disorder, coronary artery disease, myocardial infarction, and deep venous thrombosis was weak. Hence, future use of cannabis for therapeutic and recreational aims should consider its potential impact on cardiovascular diseases.

## Introduction

In United Nations drug treaties, the admission of cannabis use has been a disputed issue for some time as it induces less damage than illegal opioids. However, with the rise in more legitimate markets, *Cannabis sativa* is the most smoked substance after cigarettes, and its popularity is growing. According to statistics, in 2018, more than 192 million or 3.9% of the global adult population consumed cannabis (1). Compared with cannabis usage in low-income or middle-income countries, it is much more popular in high-income countries such as North America, Europe and Oceania (2). The prevalence of cannabis use was low but has increased in low-income and middle-income countries (3). In addition, cannabis use patterns in adults have altered in America. In the 1980s, the use of cannabis began in adolescence, with the highest consumption between the ages of 20 and 25 years, but it sharply decreased after 28 years (4). However, after 2008, adults over 30 in America frequently consumed cannabis.

Since 1996, only patients who suffer from nausea, weight loss, muscle spasm, and chronic neuropathic pain caused by multiple sclerosis were permitted to use cannabis for medical purposes in California. However, as of July 30, 2021, thirty-three American States and the District of Columbia have legalized cannabis for medicinal purposes, and the District of Columbia and eleven states have passed laws legalizing marijuana for recreational use (5). Generally, legalization will make cannabis products cheaper and more accessible for people to obtain cannabis, which is likely to increase cannabis use; in the long term, legalization may lead to an increase in marijuana-related harm (6). Although the concentration of tetrahydrocannabinol (THC) and cannabidiol (CBD) vary due to the heterogeneity of cannabis use patterns and regions, potency monitoring programs in America and Europe have shown that over the last couple of decades, THC dosage in cannabis has markedly increased from approximately 5% to more than 15%, and the mean THC: CBD ratio has also increased substantially from 23 in 2008 to 104 in 2017 (4, 6, 7). At present, even though reliable statistical data on the average THC dose are lacking, cannabis users apparently receive a higher dose of THC (8).

According to several observational clinical studies, cannabis consumption was related to various cardiovascular diseases (CVDs) such as coronary artery disease, myocardial infarction (9, 10), atrial fibrillation (11), stroke (12–15) and heart failure (16, 17). In a multicenter, interview-based study, marijuana smoking was a trigger for the onset of acute myocardial infarction. Marijuana use was associated with an increased risk of myocardial infarction onset by 4.8-fold compared to baseline non-use (10). Simple logistic regression analysis showed that teenagers (13–19 years old) who used cannabis were at a higher risk of acute myocardial infarction in a retrospective analysis using the “2012 Kids” Inpatient Database (18). The use of marijuana was also a significant risk factor for acute myocardial infarction in multiple adult-related studies, even when the patients had no other cardiac risk factors (19). An observational study based on the National Inpatient Sample Database in the USA showed that after a multivariable regression analysis of several risk factors, cannabis use remained an independent predictor of both HF and stroke in individuals between 18 and 55 years old (20). Simultaneously, according to a clinical study using a large national administrative database in the USA from 2003 to 2016, 0.5% of hospitalized teenagers (13–20 years old) with cannabis use disorder suffered arrhythmias, and the most frequent arrhythmia was atrial fibrillation (11). Furthermore, several case reports have linked marijuana smoking to atrial fibrillation (21, 22).

In epidemiological studies, however, insufficient evidence was found for CVDs because these studies included a small number of cannabis-only consumers and were not evaluated for optimal exposures. Unfortunately, most of the previous epidemiological data were short-term, retrospective and observational. As up to 70–90% of cannabis consumers smoke cigarettes simultaneously, we could not evaluate directly if differences in cannabis use caused CVD outcomes, were explained by cigarette smoking factors or were a consequence of worsening symptoms (23). As a result of simultaneous marijuana and cigarette consumption, on the basis of traditional observational studies, it was difficult to segregate the separate effects of cannabis use on CVDs. Mendelian randomization (MR) is a technique that examines the causal relationship between exposures and results by using genetic variants as instrumental variables (24). Genetic variants are inherited randomly in MR so that it can be conceptualized as a natural randomized controlled trial (25). When randomized control trials are not feasible, MR became an alternative method for exploring causal relationships between exposure and outcomes (24, 26). Recently, several studies explored the effects of substance use (cannabis use, alcohol consumption, tobacco use) on CVDs or other physical health by using the MR strategy (27–29). Multivariable Mendelian randomization is a method that incorporates genetic variants from each exposure into the same model, thus enabling simultaneous assessment of several correlated exposures (30). Multivariable MR analysis is beneficial where genetic variants are pleiotropic—that is, associated with several risk factors. Confounders that could produce erroneous relationships between exposures and outcomes can be minimized by using multivariable MR analysis. Given the potential confounding and limited causal inference obtained from observational data, we used MR and multivariable MR methods to evaluate relationships between cannabis use disorder and CVDs.

## Materials and methods

### Study design

**FIGURE 1:**
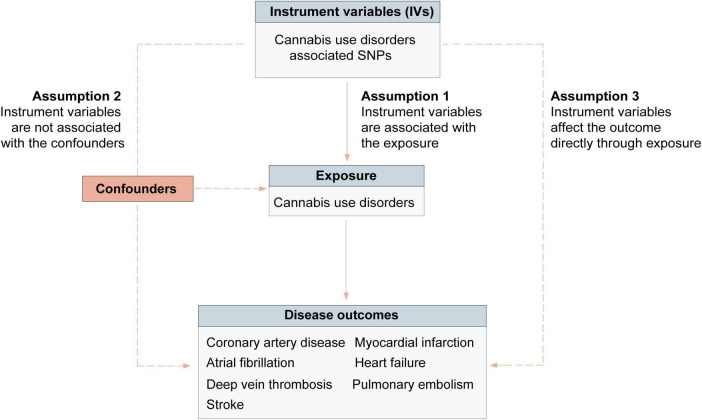
Mendelian randomization assumptions overview.

We conducted a two-sample MR analysis using summary-level data for exposures and outcomes from the publicly available genome-wide association studies (GWAS) to assess the relationships of cannabis use disorder with cardiovascular diseases. Based on three assumptions, genetic variants were used as instrumental variables (IVs) to evaluate the association between cannabis use disorder and outcomes (Figure 1). First, genetic variants are associated with the risk factor of interest (cannabis use disorder); Second, the genetic variants considered as IVs are independent from biologically plausible confounders; Third, the genetic variants should affect the outcome directly through the risk factor of interest (24). These publicly available GWAS data used in this study were presented in Supplementary Tables 1, 3. Participants in all original studies provided informed consent and ethical review approval.

### Data sources and instruments selection

#### Instrumental variables for cannabis use disorder

The GWAS summary-level data for cannabis use disorder were obtained from a GWAS meta-analysis involving 384,032 participants from Psychiatric Genomics Consortium Substance Use Disorders working group, Lundbeck Foundation Initiative for Integrative Psychiatric Research (iPSYCH), and deCODE (31). The summary statistics of European ancestry case-control individuals were used in our study (14,080 cases and 343,726 controls). The linkage disequilibrium analysis among exposure-associated SNPs was conducted in RStudio with the TwoSampleMR package, using the clump function (*r*^2^ < 0.01 and clump window was 10,000 kb) based on the 1000 genomes LD reference panel of only Europeans (CEU, TSI, FIN, GBR and IBS) (23). Only 2 SNPs at *p*-value < 5 × 10^–8^ were associated with cannabis use disorder after linkage disequilibrium analysis. We selected 12 SNPs at *p*-value < 5 × 10^–7^ associated with cannabis use disorder as instrumental variables (Supplementary Table 4). The LD Link^1^ was used to select proxies (*r*^2^ > 0.60) if no matching SNPs were available in an outcome GWAS (23). One SNP (rs72818514) for cannabis use disorder was unavailable in the pulmonary embolism GWAS, and no proxies could be used instead.

#### Genome-wide association studies summary statistics for cardiovascular outcomes

The GWAS summary statistics for coronary artery disease and myocardial infarction were obtained from the Coronary Artery Disease Genome-Wide Replication and Meta-analysis plus the Coronary Artery Disease Genetics (CardiogramplusC4D) consortium, which contained 60,801 cases (43,676 cases with myocardial infarction) and 123,504 controls (32). The majority (77%) of the participants were of European ancestry. The GWAS summary statistics of atrial fibrillation were obtained from a large GWAS meta-analysis of 65,446 cases and 522,744 controls using more than 50 studies (33). The sample was composed of 84.2% Europeans, and the data of European ancestry individuals were used in our study (55,114 cases and 482,295 controls). The GWAS summary statistics of heart failure were extracted from a recent GWAS meta-analysis containing 977,323 European participants (47,309 cases and 930,014 controls) (34). The GWAS summary statistics of stroke were obtained from a multiancestry GWAS, including 67,162 cases and 454,450 controls (35). The majority of participants were European, including 40,585 cases and 406,111 controls. We retrieved GWAS summary statistics of deep vein thrombosis and pulmonary embolism from the IEU Open GWAS Project^2^ : ukb-b, which includes GWAS summary statistics output from the GWAS pipeline using Phesant-derived variables from the UK Biobank. The sample size for deep vein thrombosis was 462,933 (9,241 cases and 453,692 controls) and pulmonary embolism was 462,933 (3,823 cases and 459,110 controls). In addition to the data for coronary artery disease and myocardial infarction being obtained from mixed populations, we used datasets of other cardiovascular diseases (atrial fibrillation, heart failure, deep vein thrombosis, pulmonary embolism, and stroke) only included participants of European descent. There was no participant overlap between the cannabis use disorder dataset and outcomes datasets. Detailed information for the GWAS of exposure and outcomes were presented in Supplementary Table 2.

Moreover, we used replication datasets of cardiovascular diseases to validate our results further. The descriptive information of replication datasets was presented in Supplementary Table 3. The summary-level data of coronary artery disease, myocardial infarction, atrial fibrillation, heart failure, deep vein thrombosis, and pulmonary embolism were derived from the UK biobank. The summary-level data for stroke were obtained from a GWAS meta-analysis of 12 case-control studies (36). The meta-analysis comprised 10,307 cases and 19,326 controls, which contained a small proportion of south Asia individuals (2,385 cases and 5,193 controls).

#### Genome-wide association studies summary statistics for risk factors

The GWAS summary data for smoking and alcohol intake were derived from a large GWAS involving 1.2 million individuals (37). We chose smoking initiation phenotypes as our instrument for smoking, which indicated whether an individual had ever smoked regularly. Alcohol intake was measured with drinks per week. The GWAS summary data for body mass index were obtained from a large GWAS meta-analysis involving 339,224 individuals (38). Body mass index was measured or self-reported weight in kg per height in meters squared. The data for blood lipid, including low-density lipoprotein cholesterol, high-density lipoprotein cholesterol, triglycerides, and total cholesterol, was obtained from a GWAS meta-analysis containing 94,595 European individuals (39). The summary data for type 2 diabetes was obtained from a GWAS meta-analysis, including 26,488 cases and 83,964 controls (40). The summary statistics of hypertension were derived from the UK biobank, which contained 463,010 individuals. The summary data for depression were extracted from the largest GWAS mate-analysis containing 807,553 individuals (excluding 23andme data because of restricted access) (41).

### Statistical analysis

**FIGURE 2:**
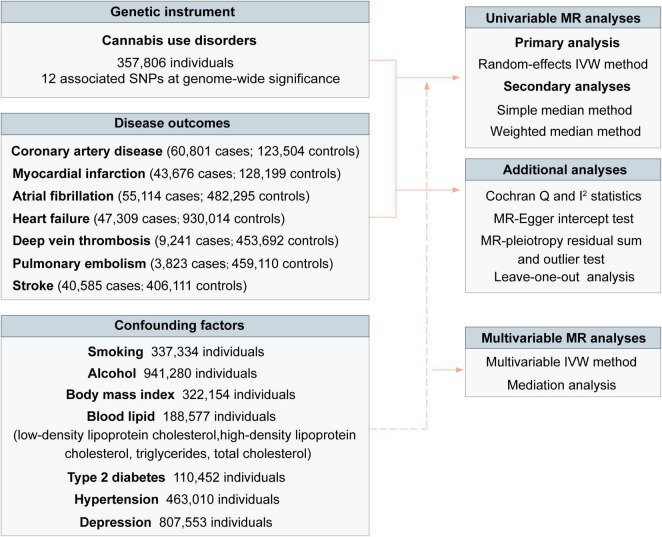
Overview of study design. IVW indicated the inverse-variance weighted method.

The workflow of performed analyses was presented in Figure 2. For each significant SNP, the *R*^2^ was calculated as follows: (2 × EAF × (1-EAF) × beta^2^)/(2 × EAF × (1-EAF) × beta^2^ + 2 × EAF × (1-EAF) × N × se^2^) (42), where EAF was the effect allele frequency, N was the sample size, and beta was the estimated effect on cannabis use disorder. The F-statistic was calculated to estimate the strength of genetic instruments using the formula: F-statistic = *R*^2^ × (*N* - 2)/(1 - *R*^2^) (42). The *R*^2^ and *F*-statistics for each SNP were presented in Supplementary Table 4. We used the online tool^3^ to calculate *a priori* statistical power. The 12 SNPs for cannabis use disorder explained an estimated 0.1% of phenotypic variability. Power estimates for the 12 SNPs of cannabis use disorder classified by outcomes were presented in Supplementary Table 5.

Following the extraction of estimates and the harmonization of estimates via the effect alleles, we used the Wald Estimator to generate main effect estimates and the Delta method to calculate standard error (43). The MR estimates were combined as standard analysis using the multiplicative random-effects inverse-variance weighted (IVW) method (44). We pooled estimates using complementary simple and weighted median methods as a sensitivity analysis. When at least 50% of the weight is derived from valid instrumental variables, the median method can generate consistent estimates (44). The Cochran Q and *I*^2^ statistics were performed to assess heterogeneity among estimates across individual SNPs (44, 45). Heterogeneity was considered if the *p*-value < 0.05 and *I*^2^ were used to quantify heterogeneity (*I*^2^ ≤ 25%: low heterogeneity; 25% < *I*^2^ ≤ 50%: moderate heterogeneity; *I*^2^ ≥ 50%: high heterogeneity). A random-effects model was more suitable if the heterogeneity was high. We also performed the MR-Egger intercept test to investigate horizontal pleiotropy (44, 46). The MR-pleiotropy residual sum and outlier test was adopted to detect and correct for horizontal pleiotropic outliers in the IVW method (SNPs > 10) (47). The detected outliers will be excluded and corrected. Furthermore, we conducted a leave-one-out sensitivity analysis to preclude the possibility that the causal inference was driven by a single SNP. Multivariable MR analyses were performed to evaluate the direct effect of cannabis use disorder on CVD outcomes whilst accounting for potential mediation effects by smoking, alcohol, body mass index, blood lipid, type 2 diabetes, and hypertension, which were the common cardiovascular risk factors (48). Given the strong genetic correlation between cannabis use disorder and depression (31), we also conducted a multivariable MR analysis adjusting for genetic liability to depression. Confounders were subsequently explored via mediation analysis, as previously described, to estimate the mediation effects on the pathway from cannabis use disorder to CVDs (49, 50).

A *p*-value < 0.05 was considered statistically significant. We adjusted the *p*-value by performing FDR correction (*q* value) using the Benjamini-Hochberg method with an FDR threshold *q* < 0.05. MR analyses were conducted using the TwoSampleMR (version 0.5.5), MendelianRandomization (version 0.4.3), MVMR (version 0.3) and MRPRESSO (version 1.0) in R. All data analyses were conducted with R version 3.6.1.

## Results

### Association of genetic liability to cannabis use disorder with cardiovascular diseases

**FIGURE 3:**
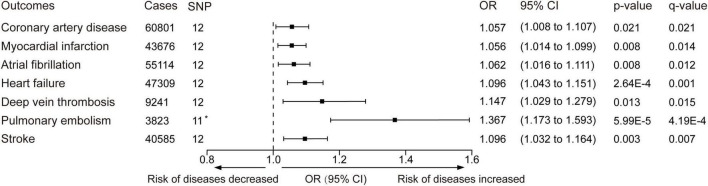
Inverse-variance–weighted Mendelian randomization was performed to determine whether the genetic liability to cannabis use disorder is related to cardiovascular disease. OR, odds ratio. Estimates and *p*-value were from the random-effect inverse variance-weighted method. CI, confidence interval. The *q*-values represent Benjamini-Hochberg’s FDR-corrected *p*-value. The horizontal line represented the odds ratio and 95% confidence interval, OR < 1.0 indicated the risk of diseases decreased and OR > 1.0 indicated the risk of diseases increased. *One SNP (rs72818514) was excluded in the pulmonary embolism outcome because no available proxy was found.

The minimum F statistic of the genetic variants was 25.5. The standard IVW analyses showed that cannabis use disorder was positively associated with several cardiovascular diseases (Figure 3 and Supplementary Figure 1). The odds ratios were 1.057 (95% CI: 1.008–1.107, *p*-value = 0.021, *q*-value = 0.021) for coronary artery disease, 1.056 (95% CI: 1.014–1.099, *p*-value = 0.008, *q*-value = 0.014) for myocardial infarction, 1.062 (95% CI: 1.016–1.111, *p*-value = 0.008, *q*-value = 0.012) for atrial fibrillation, 1.096 (95% CI: 1.043–1.151, *p*-value = 2.64 × 10^–4^, *q*-value = 0.001) for heart failure 1.147 (95% CI: 1.029–1.279, *p*-value = 0.013, *q*-value = 0.015) for deep vein thrombosis, 1.367 (95% CI: 1.173–1.593, *p*-value = 5.99 × 10^–5^, *q*-value = 4.19 × 10^–4^) for pulmonary embolism, and 1.096 (95% CI: 1.032–1.164, *p*-value = 0.003, *q*-value = 0.007) for stroke. The IVW estimates and 95% CIs were broadly consistent with estimates from the simple median and weighted median sensitivity analyses (Supplementary Table 6). Nevertheless, except for stroke, the *q*-value of coronary artery disease, myocardial infarction, atrial fibrillation, heart failure, deep vein thrombosis, and pulmonary embolism failed to reach statistical significance in the sensitivity analyses (Supplementary Table 6).

There was no heterogeneity for cannabis use disorder with coronary artery disease, myocardial infarction, or atrial fibrillation. However, there was slight heterogeneity for cannabis use disorder with heart failure (*I*^2^ = 2.7%, *P* = 0.416) and pulmonary embolism (*I*^2^ = 7.4%, *p*-value = 0.377), and moderate heterogeneity for cannabis use disorder with deep vein thrombosis (*I*^2^ = 28.1%, *p*-value = 0.171) and stroke (*I*^2^ = 30.4%, *p*-value = 0.149) (Supplementary Table 7). Similarly, the MR-Egger intercept analysis did not detect directional pleiotropy (Supplementary Table 7). No outlier SNPs were detected with the MR-pleiotropy residual sum and outlier test (Supplementary Table 6). The leave-one-out sensitivity analysis revealed that no single SNP influenced the IVW estimate for each outcome except coronary artery disease. Three SNPs (rs12536335, rs1392816, and rs55986679) influenced the estimate for coronary artery disease (Supplementary Figure 2). We further analyzed the effect of cannabis use disorders on cardiovascular diseases by using replication datasets. The effect of cannabis use disorders on atrial fibrillation, deep vein thrombosis, pulmonary embolism and stroke were robust. However, the association between cannabis use disorders and coronary artery disease, myocardial infarction, and heart failure was not positive when using datasets with smaller sample numbers (Supplementary Table 8).

We also evaluated the effects of those CVDs on cannabis use disorder. However, genetic ability to CVDs was not associated with cannabis use disorder (Supplementary Table 9).

### Multivariable Mendelian randomization analysis

In the multivariable MR analysis adjusting for common cardiovascular risk factors (smoking, alcohol, body mass index, blood lipid, type 2 diabetes, hypertension) and depression, the overall patterns for the associations of cannabis use disorder with atrial fibrillation, heart failure, pulmonary embolism, and stroke remained (Table 1). The association with deep venous thrombosis did not persist in the multivariable MR analysis adjusting for smoking, alcohol, hypertension, and depression. Likewise, the pattern of the association between cannabis use disorder with coronary artery disease and myocardial infarction did not persist in the multivariable MR analysis adjusting for cardiovascular risk factors and depression. Mediation analysis were subsequently conducted to investigate the mediation effects of those confounders (Supplementary Table 10). The mediation analysis results showed that smoking, body mass index, low-density lipoprotein, hypertension, and depression have more significant mediation effects than other confounders, which suggested these factors might partly mediate the link from cannabis use disorder to these CVDs.

**TABLE 1:** Multivariable Mendelian randomization associations of cannabis use disorder with cardiovascular diseases adjusting for risk factors.

Model	Outcomes OR (95% CI)						
	CAD	MI	AF	HF	DVT	PE	Stroke
Unadjusted	1.057 (1.008, 1.107) *p* = 0.021	1.056 (1.014, 1.099) *p* = 0.008	1.062 (1.016, 1.111) *p* = 0.008	1.096 (1.043, 1.151) *p* = 2.64E-04	1.147 (1.029, 1.279) *p* = 0.013	1.367 (1.173, 1.593) *p* = 5.99E-05	1.065 (1.024, 1.107) *p* = 0.003
Smoking	1.018 (0.941, 1.102) *p* = 0.651	1.017 (0.931, 1.110) *p* = 0.707	1.066 (1.009, 1.125) *p* = 0.022	1.087 (1.015, 1.164) *p* = 0.017	1.041 (0.919, 1.177) *p* = 0.531	1.247 (1.017, 1.530) *p* = 0.034	1.097 (1.006, 1.197) *p* = 0.036
Alcohol	1.062 (0.990, 1.138) *p* = 0.094	1.053 (0.975, 1.139) *p* = 0.187	1.055 (0.998, 1.115) *p* = 0.061	1.114 (1.050, 1.182) *p* = 3.18E-04	1.077 (0.958, 1.210) *p* = 0.214	1.459 (1.219, 1.747) *p* = 3.979E-05	1.059 (0.992, 0.123) *p* = 0.085
BMI	1.059 (0.990, 1.132) *p* = 0.093	1.048 (0.973, 1.127) *p* = 0.214	1.067 (1.013, 1.123) *p* = 0.013	1.088 (1.026, 1.154) *p* = 0.005	1.161 (1.023, 1.317) *p* = 0.021	1.332 (1.121, 1.584) *p* = 0.001	1.094 (1.016, 1.178) *p* = 0.017
HDL	1.055 (0.989, 1.125) *p* = 0.104	1.051 (0.979, 1.130) *p* = 0.166	1.062 (1.011, 1.116) *p* = 0.017	1.090 (1.031, 1.151) *p* = 0.002	1.197 (1.069, 1.342) *p* = 0.002	1.357 (1.143, 1.610) *p* = 4.55E-04	1.111 (1.034, 1.194) *p* = 0.004
LDL	1.037 (0.965, 1.113) *p* = 0.331	1.045 (0.966, 1.132) *p* = 0.273	1.077 (1.019, 1.137) *p* = 0.009	1.104 (1.034, 1.178) *p* = 0.003	1.197 (1.047, 1.369) *p* = 0.009	1.380 (1.139, 1.672) *p* = 0.009	1.122 (1.039, 1.210) *p* = 0.003
TC	1.031 (0.960, 1.107) *p* = 0.399	1.036 (0.957, 1.121) *p* = 0.391	1.074 (1.016, 1.133) *p* = 0.011	1.125 (1.061, 1.195) *p* = 8.38E-05	1.163 (1.016, 1.331) *p* = 0.028	1.412 (1.172, 1.699) *p* = 2.82E-04	1.131 (1.052, 1.214) *p* = 0.001
TG	1.041 (0.968, 1.120) *p* = 0.278	1.030 (0.951, 1.117) *p* = 0.462	1.058 (1.004, 1.114) *p* = 0.035	1.093 (1.023, 1.168) *p* = 0.008	1.165 (1.015, 1.339) *p* = 0.030	1.303 (1.081, 1.570) *p* = 0.005	1.117 (1.030, 1.213) *p* = 0.007
T2D	1.044 (0.979, 1.113) *p* = 0.191	1.038 (0.966, 1.114) *p* = 0.311	1.065 (1.013, 1.119) *p* = 0.013	1.106 (1.043, 1.174) *p* = 7.61E-04	1.176 (1.040, 1.330) *p* = 0.010	1.331 (1.126, 1.571) *p* = 7.66E-04	1.106 (1.029, 1.188) *p* = 0.006
hypertension	1.046 (0.978, 1.117) *p* = 0.188	1.049 (0.974, 1.129) *p* = 0.206	1.053 (1.002, 1.106) *p* = 0.041	1.100 (1.038, 1.164) *p* = 0.001	1.112 (0.983, 1.257) *p* = 0.091	1.435 (1.184, 1.737) *p* = 2.30E-04	1.061 (1.005, 1.121) *p* = 0.032
Depression	1.031 (0.942, 1.129) *p* = 0.506	1.053 (0.953, 1.164) *p* = 0.306	1.087 (1.010, 1.169) *p* = 0.025	1.106 (1.018, 1.202) *p* = 0.017	1.035 (0.876, 1.221) *p* = 0.688	1.314 (1.009, 1.711) *p* = 0.043	1.094 (1.017, 1.176) *p* = 0.016

CAD, Coronary artery disease; MI, myocardial infarction; AF, atrial fibrillation; HF, heart failure; DVT, deep vein thrombosis; PE, pulmonary embolism; BMI, body mass index; HDL, high-density lipoprotein cholesterol; LDL, low-density lipoprotein cholesterol; TG, triglycerides; TC, total cholesterol; OR, odds ratio; CI, confidence interval.

## Discussion

Throughout the last couple of decades, emerging data have proposed a relationship between cannabis use and the risk of CVDs such as myocardial infarction (9), cardiomyopathy, arrhythmias (21, 22) and cardiac arrest (5, 51). However, prior observational clinical studies may be restricted to unexplained confounding factors (tobacco use) (31), reverse causality (52), and measurement error (inaccurate memory or society’s expectations) (17). In an *in vivo* animal study, the protective effect of CBD was illustrated in myocardial infarction, stroke, doxorubicin-induced and diabetic cardiomyopathies, and autoimmune myocarditis (16, 53–56). Further clarification of whether cannabis use increases the risk of cardiovascular outcomes may promote the regulated and scientific use of cannabis. Therefore, we performed an MR analysis using SNPs reported to be associated with cannabis use disorder to investigate the connection of cannabis use disorder on the risk of cardiovascular outcomes.

Our MR results provided clues for the relationship between cannabis use disorder and several cardiovascular outcomes. These results aroused our curiosity and interest in cannabis use. Even after adjusting for several common cardiovascular risk factors in multivariable MR analysis, the overall patterns for the associations of cannabis use disorder with atrial fibrillation, heart failure, pulmonary embolism, and stroke remained. The mediation analysis results suggested that smoking, body mass index, low-density lipoprotein, hypertension, and depression might partly mediate the link from cannabis use disorder to coronary artery disease, myocardial infarction, and deep venous thrombosis. Cannabis consumption was often accompanied by smoking. It was not surprising that the mediation effect of smoking was substantial. Cannabis use was also related to depression development in adolescents (57). However, there was no strong evidence for the association between cannabis use, body mass index, blood lipid, and hypertension (58–60). The results of the IVW analysis were inconsistent with the results in the simple and weighted median sensitivity analyses. The inconsistency of the estimates from different methods suggested that the genome-wide significant SNPs for cannabis use disorder are not all valid instrumental variables (*p*-value of most SNPs > 5 × 10^–8^) (44), although the F-statistics were all greater than 10. A survey reported that over 2 million Americans with diagnosed cardiovascular diseases currently consumed or have consumed cannabis (17). The genetic liability to CVDs may cause heavier cannabis use; thus, we evaluated the effects of these CVDs on cannabis use disorder. However, no evidence was found for the genetic liability to CVDs with cannabis use disorder. The result demonstrated no reverse causality, which supported the causal interpretation.

The latest study investigated the association between lifetime cannabis use and cardiovascular diseases by using MR analysis (61). Their results did not indicate a causal effect of genetic predisposition to lifetime cannabis use on several cardiovascular diseases. Lifetime cannabis use was defined as any use of cannabis during the lifetime, even if only used once or was a long time ago (62); however, cases in the GWAS of cannabis use disorder met the criteria for cannabis abuse or dependence (31), so cannabis use disorder was regarded as an exposure reflecting heavy lifetime use (23). It was reasonable to believe that the cannabis use disorder phenotype reflected more cannabis use than the lifetime cannabis use phenotype. Hence, we considered that the discrepancy between lifetime cannabis use and cannabis use disorder on cardiovascular diseases might be due to the difference in exposure time. The genetic liability to cannabis use and cannabis use disorder were also distinguished (31). Besides, the composition of the plant and the route of administration influenced the cardiovascular effects of cannabis (16).

Several studies, including case reports, have described the relationship between the cardiac electrophysiologic effect and marijuana use, including atrial fibrillation/atrial flutter, atrioventricular block, sick sinus syndrome, ventricular tachycardia, and Brugada pattern (11, 22, 63, 64). In theory, THC stimulation would increase the content of catecholamines and b-adrenaline in cardiac tissue, which may promote arrhythmogenesis. In both rabbit and dog ventricular papillary muscle, CBD increased action potential duration, decreased the rapid delayed rectifier potassium currents, the slow delayed rectifier potassium currents and the transient outward rectifier potassium currents (65). Consistent with clinical studies, the MR analysis showed that cannabis use disorder was causally associated with atrial fibrillation; however, lifetime cannabis use had no causality with atrial fibrillation. According to our analysis and the clinical observational study, the electrophysiological effect of cannabis use disorder should be considered carefully. However, in medical use, occasional marijuana use did not affect the incidence of atrial fibrillation.

Evidence from a previous observational study indicated that patients with cannabis use disorder have significantly higher incidence of venous thromboembolism, deep vein thromboses, and pulmonary embolism, consistent with our results (66). These associations may be supported by some mechanistic findings. Long-term cannabis use can lead to the deterioration of hematopoietic cells and change red cell indices, which are risk factors for venous thromboembolism (67, 68). A vitro study showed that THC could lead to platelet aggregation and Factor VII activation, which facilitate the process of coagulation (69).

A longitudinal cohort study based on a general population survey of Australians aged 20–24 years, 40–44 years and 60–64 years revealed that compared with non-cannabis users, the rate of stroke/transient ischemic attack in heavy cannabis users was 3.3 times higher than in non-cannabis users, after adjusting for age. Following adjustment for covariates such as tobacco smoking, the rate of non-fatal strokes or transient ischemic attacks among those who used cannabis weekly was higher than that of non-users (12). According to Behavioral Risk Factor Surveillance System Survey Analysis, the odds of stroke for young adults who had recently used marijuana were 1.82 times higher than those without recent marijuana use and 2.45 times higher for frequent marijuana users (> 10 days/month) (70). A longitudinal cohort study in parous women also revealed that cannabis use disorders might increase the long-term risk of CVDs in women, particularly hemorrhagic stroke (15). In addition, our study provided evidence that genetically determined cannabis use disorder had a detrimental effect on stroke. In rats, short-term exposure to secondhand marijuana smoke significantly impaired endothelial function for at least one hour. The duration of such impairment was significantly longer than comparable impairment caused by secondhand smoke (71). A prospective study of 48 consecutive young patients showed that multifocal intracranial stenosis was related to cannabis use in 21% of patients, which suggested that multifocal angiopathy related to cannabis consumption may be a significant cause of ischemic stroke in young people (72).

It is essential to consider how to interpret a causal effect estimate of a binary exposure when performing two-sample MR. The legal status of cannabis makes cannabis exposure uncommon, so the effect of the exposure cannot always be attributed to the exposure itself. In GWAS of cardiovascular diseases, individuals may carry the risk allele but have never been exposed to cannabis. Under the circumstances, the causal effect estimate should be interpreted as the effect of genetic liability to cannabis (23).

## Strengths, limitations, and prospection

Our study has several strengths. In contrast to observational studies, MR analysis, particularly multivariable MR analysis, reduce the bias that may occur in observational studies due to confounders. Because of the legal status of cannabis, it is hard to conduct cannabis-related studies. The present MR study leveraged population-scale human genetics to support evidence for a causal association between cannabis use disorder and several cardiovascular diseases, which is a supplement to the existing research. We only included participants of European descent in the exposure and outcome datasets (except for coronary artery disease and myocardial infarction), which can reduce the population stratification bias.

There are some limitations in our study. First, the statistical power of our study seemed inadequate to determine the effects of cannabis use on cardiovascular health. The availability of more extensive GWAS of cannabis use disorder in the future will enhance the accuracy of our MR estimates by providing more exposure variables. Second, the results from some sensitivity analyses were inconsistent with the main findings, which suggested that our results were unstable and may be biased by horizontal pleiotropy. Third, we performed LD analysis using the 1000 Genomes panel of Europeans as the LD reference, but LD could be different in populations even in one ancestry. Fourth, cannabis usually produces deleterious effects based on potency, which is influenced by the amount of psychoactive THC in it. However, our MR study did not examine the differential health effects of THC and the potential offset due to the co-administration of CBD on different diseases. Moreover, genetic variants used as instruments may vary over age in their relationship with the exposure, leading to a risk of bias in MR analyses. We considered this bias in our MR analyses minor for some reasons: First, the exposures in this study were binary exposure variables. Cannabis use disorder included cannabis abuse or dependence that reflected heavy lifetime use. These phenotypes may change small over time. Besides, cannabis use often starts in the middle to late teenage years and peaks in the early and middle 20s. After employment, marriage, and having children, cannabis use declines steeply (73). The bias tended to decrease when exposure windows were short (74).

The levels of evidence from previous observative studies have not been robust, and the present study provides novel additional evidence. The cannabis plant contains more than 60 compounds with varying pharmacological properties (75). More research should be conducted to investigate the different effects of those compounds so that safe and effective products can be developed. Moreover, the GWAS of biomarkers of cannabis exposure, such as 11-nor-Δ9-tetrahydrocannabinol-9-carboxylic acid or DNA methylation markers could also be used in further MR studies (76).

## Conclusion

The present MR study supported a potentially causal association between cannabis use disorder with higher risks of atrial fibrillation, heart failure, pulmonary embolism, and stroke. The evidence for the association between cannabis use disorder, coronary artery disease, myocardial infarction, and deep venous thrombosis was weak. Additional studies, especially clinical studies, are required to verify the effects of cannabis on cardiovascular. Our results suggest caution and alertness for potential public health hazards in cannabis use.

## Data availability statement

The datasets presented in this study can be found in online repositories. The names of the repository/repositories and accession number(s) can be found in the article/Supplementary material.

## Author contributions

ZW and LM conceived and designed the experiments. MC and X-FC performed the experiments, analyzed the data, and revised manuscript. Y-LL wrote the manuscript. All authors read and approved the manuscript.

## Footnotes

1
https://analysistools.cancer.gov/LDlink

2
https://gwas.mrcieu.ac.uk

3
https://shiny.cnsgenomics.com/mRnd/
