# Supplementary material for: Association of cannabis use disorder with cardiovascular diseases: A two-sample Mendelian randomization study
Source: Front Cardiovasc Med. 2022 Oct 6;9:966707. doi: 10.3389/fcvm.2022.966707 (PMC9582269; doi:10.3389/fcvm.2022.966707)
Supplement: Supplementary file 1 [file Data_Sheet_1.PDF]

Association of cannabis use disorder with cardiovascular diseases： A two-sample Mendelian randomization study

Miao Chen, Yun-long Lu, Zhen Wang, Liang Ma

Supplementary Material

Table 1. Descriptive information of the datasets included in the analyses.

GWAS	Phenotype	Participants	Ancestry	Used in MR	ID in IGD*	Web link for data source
Johnson EC, 2020	Cannabis use disorder	14,080 cases<br>343,726 controls	European	Exposure	—	https://www.med.unc.edu/pgc/download-results/
GSCAN	Smoking	337,334 individuals	European	Confounder	—	https://www.ebi.ac.uk/gwas/publications/30643251
GSCAN	Alcohol	941,280 individuals	European	Confounder	—	https://www.ebi.ac.uk/gwas/publications/ 30643251
GIANT	Body mass index	322,154 individuals	European	Confounder	ieu-a-835	https://gwas.mrcieu.ac.uk/
GLGC	Blood lipid	188,577 individuals	European	Confounder	ieu-a-299, ieu-a-300<br>ieu-a-301, ieu-a-302	https://gwas.mrcieu.ac.uk/
DIAGRAM	Type 2 diabetes	110,452 individuals	Multi-ancestry	Confounder	ieu-a-23	https://gwas.mrcieu.ac.uk/
UK biobank (MRC-IEU, 2018)	Hypertension	463,010 individuals	European	Confounder	ukb-b-12493	https://gwas.mrcieu.ac.uk/
Howard et al, 2019	Depression	246,363 cases<br>561,190 controls	Multi-ancestry	Confounder	—	https://datashare.ed.ac.uk/handle/10283/3203
CARDIoGRAMplusC4D	Coronary artery disease	60,801 cases<br>123,504 controls	Multi-ancestry	Outcome	ieu-a-7	https://gwas.mrcieu.ac.uk/
CARDIoGRAMplusC4D	Myocardial infarction	43,676 cases<br>128,199 controls	Multi-ancestry	Outcome	ieu-a-798	https://gwas.mrcieu.ac.uk/
Roselli C, 2018	Atrial fibrillation	55,114 cases<br>482,295 controls	European	Outcome	ebi-a-GCST006061	https://gwas.mrcieu.ac.uk/
Shah S, 2020	Heart failure	47,309 cases<br>930,014 controls	European	Outcome	—	https://cvd.hugeamp.org/downloads.html

UK biobank (MRC-IEU, 2018)	Deep vein thrombosis	9,241 cases 453,692 controls	European	Outcome	ukb-b-12040	https://gwas.mrcieu.ac.uk/
UK biobank (MRC-IEU, 2018)	Pulmonary embolism	3,823 cases 459,110 controls	European	Outcome	ukb-b-16048	https://gwas.mrcieu.ac.uk/
Malik R, 2018	Stroke	40,585 cases 406,111 controls	European	Outcome	ebi-a-GCST005838	https://gwas.mrcieu.ac.uk/

*IGD: The IEU GWAS database (https://gwas.mrcieu.ac.uk/)

Table 2. Detailed information for the genome-wide association study of exposure and outcomes.

GWAS	Cases source	Cases definition
Cannabis use disorder	Psychiatric Genomics Consortium Substance Use Disorders working group, Lundbeck Foundation Initiative for Integrative Psychiatric Research, and deCODE	DSM-IV (or DSM-III-R) cannabis abuse or dependence, DSM-5 cannabis use disorder, ICD-10 codes of F12.1 (cannabis abuse) or F12.2 (cannabis dependence).
Coronary artery disease, Myocardial infarction	Coronary Artery Disease Genome-Wide Replication and Meta-analysis and Coronary Artery Disease Genetics (CardiogramplusC4D) consortium	An inclusive CAD diagnosis of MI, acute coronary syndrome, chronic stable angina or coronary stenosis of >50%
Atrial fibrillation	AFGen consortium, Broad AF study, UK Biobank, and Biobank Japan	Participants with paroxysmal or permanent AF, or atrial Flutter. Codes in UK biobank: non-cancer illness code, self-reported (1471, 1483); operation code (1524); diagnoses-main/secondary ICD10 (I48, I48.0-4, I48.9); underlying (primary/secondary) cause of death: ICD10 (I48, I48.0-4, I48.9); diagnoses-main/secondary ICD9 (4273); operative procedures-main/secondary OPCS (K57.1, K62.1-4)
Heart failure	26 cohorts with either a case-control or population-based study design	A clinical diagnosis of HF of any aetiology with no inclusion criteria based on LV ejection fraction
Deep vein thrombosis	UK Biobank	Code in UK biobank: non-cancer illness code, self-reported (deep venous thrombosis)
Pulmonary embolism	UK Biobank	Code in UK biobank: non-cancer illness code, self-reported (pulmonary embolism +/- dvt)
Stroke	29 studies	Defined according to the World Health Organization (WHO), signs of rapid development of focal (or global) disturbance of cerebral function, and lasting more than 24 hours or leading to death with no apparent cause other than that of vascular origin

Table 3. Descriptive information of the replication datasets of cardiovascular diseases used in the analyses

GWAS	Phenotype	Participants	Ancestry	ID in IGD*	Web link for data source
UK biobank (Neale lab, 2018)	Coronary artery disease	10,157 cases 351,037 controls	European	ukb-d-I9_CHD	https://gwas.mrcieu.ac.uk/
UK biobank (MRC-IEU, 2018)	Myocardial infarction	10,616 cases 452,317 controls	European	ukb-b-15829	https://gwas.mrcieu.ac.uk/
Nielsen JB, 2018	Atrial fibrillation	60,620 cases 970,216 controls	European	ebi-a-GCST006414	https://gwas.mrcieu.ac.uk/
UK biobank (Neale lab, 2018)	Heart failure	1,405 cases 359,789 controls	European	ukb-d-HEARTFAIL	https://gwas.mrcieu.ac.uk/
UK biobank (Neale Lab, 2017)	Deep vein thrombosis	6,767 cases 330,392 controls	European	ukb-a-65	https://gwas.mrcieu.ac.uk/
UK biobank (Neale Lab, 2017)	Pulmonary embolism	2,801 cases 334,358controls	European	ukb-a-64	https://gwas.mrcieu.ac.uk/
Malik, 2016	Stroke	10,307 cases 19,326 controls	Mixed	ieu-a-1108	https://gwas.mrcieu.ac.uk/

*IGD: The IEU GWAS database (https://gwas.mrcieu.ac.uk/)

Table 4. Associations of single nucleotide polymorphisms for cannabis use disorder.

SNP	Chr	Position	EA	NEA	EAF*	Beta	SE	P-value	N	R^2	F-statistic
rs1392816	1	66481188	T	C	0.384	-0.0998	0.0184	6.14E-08	351174	8.38E-05	29.4
rs1509378	2	22754466	A	G	0.334	0.0963	0.0189	3.40E-07	351090	7.39E-05	26.0
rs2624841	3	50198415	T	C	0.225	0.0897	0.0176	3.42E-07	355892	7.30E-05	26.0
rs72818514	5	160078222	T	C	0.056	-0.1828	0.0342	9.33E-08	355548	8.03E-05	28.6
rs553920	6	53442069	T	C	0.238	0.1040	0.0198	1.60E-07	353969	7.79E-05	27.6
rs12536335	7	114043159	A	G	0.548	0.0916	0.0171	8.12E-08	355646	8.07E-05	28.7
rs55986679	8	27406353	A	T	0.884	0.1262	0.0216	4.81E-09	356967	9.56E-05	34.1
rs10886017	10	118672531	A	C	0.243	0.0990	0.0195	3.68E-07	353710	7.29E-05	25.8
rs3914059	11	113503954	T	C	0.337	-0.0850	0.0168	3.89E-07	356914	7.17E-05	25.6
rs9787909	11	28375949	A	C	0.826	0.1137	0.0225	4.52E-07	354449	7.20E-05	25.5
rs17271123	15	62100506	T	G	0.406	0.1284	0.0252	3.54E-07	291017	8.92E-05	26.0
rs590076	18	53260732	A	G	0.342	0.0874	0.0171	3.11E-07	357031	7.32E-05	26.1

SNP, single nucleotide polymorphism; Chr, chromosome; EA, effect allele; NEA, non-effect allele; EAF, effect allele frequency.

*EAF were obtained from the ALFA project (https://www.ncbi.nlm.nih.gov/snp)

Table 5. Sample size and power calculations in Mendelian randomization study of cannabis use disorder and risk of cardiovascular.

Outcome	Sample size	Proportion of cases	Selected scenarios*					
			OR=1.10	OR=1.20	OR=1.30	OR=1.40	OR=1.50	OR=1.60
Coronary artery disease	184305	0.33	0.09	0.23	0.42	0.63	0.80	0.91
Myocardial infarction	171875	0.25	0.09	0.19	0.36	0.55	0.73	0.86
Atrial fibrillation	537409	0.10	0.11	0.28	0.53	0.77	0.92	0.98
Heart failure	977323	0.05	0.10	0.27	0.52	0.76	0.92	0.98
Deep vein thrombosis	462933	0.02	0.06	0.09	0.15	0.22	0.32	0.43
Pulmonary embolism	462933	0.01	0.06	0.07	0.10	0.14	0.19	0.25
Stroke	446696	0.09	0.09	0.22	0.43	0.65	0.83	0.94

*Type 1 error of 5% and a proportion of variance explained equal to 4% are assumed.

OR: True odds ratio of the outcome variable per standard deviation of the exposure variable.

Table 6. Estimates for the association between cannabis use disorder and cardiovascular diseases.

Outcome	Method	OR	95% CI	P-value	Q-value
Coronary artery disease	IVW (multiplicative random effects)	1.057	(1.008, 1.107)	0.021	0.021
	Simple median	1.062	(0.983, 1.147)	0.128	0.179
	Weighted median	1.052	(0.970, 1.140)	0.219	0.256
	MR PRESSO			0.790	
Myocardial infarction	IVW (multiplicative random effects)	1.056	(1.014, 1.099)	0.008	0.014
	Simple median	1.039	(0.960, 1.124)	0.341	0.341
	Weighted median	1.019	(0.937, 1.109)	0.659	0.659
	MR PRESSO			0.963	
Atrial fibrillation	IVW (multiplicative random effects)	1.062	(1.016, 1.111)	0.008	0.012
	Simple median	1.066	(1.004, 1.131)	0.035	0.082
	Weighted median	1.059	(0.996, 1.126)	0.067	0.156
	MR PRESSO			0.489	
Heart failure	IVW (multiplicative random effects)	1.096	(1.043, 1.151)	2.64E-04	0.001
	Simple median	1.067	(0.999, 1.140)	0.053	0.094
	Weighted median	1.061	(0.991, 1.136)	0.089	0.155
	MR PRESSO			0.431	
Deep vein thrombosis	IVW (multiplicative random effects)	1.147	(1.029, 1.279)	0.013	0.015
	Simple median	1.104	(0.967, 1.259)	0.143	0.166
	Weighted median	1.116	(0.978, 1.274)	0.104	0.146
	MR PRESSO			0.194	
Pulmonary embolism	IVW (multiplicative random effects)	1.367	(1.173, 1.593)	5.99E-05	4.19E-04
	Simple median	1.304	(1.053, 1.615)	0.015	0.052
	Weighted median	1.289	(1.043, 1.593)	0.019	0.065
	MR PRESSO			0.422	
Stroke	IVW (multiplicative random effects)	1.096	(1.032, 1.164)	0.003	0.007
	Simple median	1.113	(1.036, 1.196)	0.003	0.024
	Weighted median	1.110	(1.033, 1.194)	0.005	0.033
	MR PRESSO			0.180	

Table 7. Heterogeneity and MR-Egger test for Horizontal pleiotropy

Exposure	Outcomes	Heterogeneity			
		Q	O_df	P	I^2 (%)
Cannabis use disorder	Coronary artery disease	7.0	11	0.797	0
	Myocardial infarction	4.3	11	0.962	0
	Atrial fibrillation	11.0	11	0.440	0
	Heart failure	11.3	11	0.416	2.7
	Deep vein thrombosis	15.3	11	0.171	28.1
	Pulmonary embolism	10.8	10	0.377	7.4
	Stroke	15.8	11	0.149	30.4
		MR-Egger test for directional pleiotropy			
		Egger_intercept	Standard error	P	
Cannabis use disorder	Coronary artery disease	0.007	0.019	0.699	
	Myocardial infarction	-0.006	0.021	0.782	
	Atrial fibrillation	0.003	0.014	0.324	
	Heart failure	0.010	0.015	0.512	
	Deep vein thrombosis	-9.10E-04	0.034	0.979	
	Pulmonary embolism	0.012	0.060	0.849	
	Stroke	-0.003	0.020	0.869	

Table 8. Estimates for the association between cannabis use disorder and cardiovascular diseases (use replication datasets of cardiovascular diseases).

Outcome	Method	OR	95% CI	P-value	Q-value
Coronary artery disease	IVW (multiplicative random effects)	1.035	(0.900, 1.191)	0.626	0.730
	Simple median	1.100	(0.958, 1.263)	0.178	0.249
	Weighted median	1.077	(0.937, 1.238)	0.298	0.417
Myocardial infarction	IVW (multiplicative random effects)	1.074	(0.955, 1.208)	0.235	0.329
	Simple median	1.067	(0.935, 1.218)	0.338	0.394
	Weighted median	1.044	(0.910, 1.199)	0.538	0.628
Atrial fibrillation	IVW (multiplicative random effects)	1.068	(1.016, 1.123)	0.010	0.017
	Simple median	1.086	(1.023, 1.152)	0.007	0.024
	Weighted median	1.045	(0.981, 1.114)	0.170	0.298
Heart failure	IVW (multiplicative random effects)	1.051	(0.843, 1.310)	0.657	0.657
	Simple median	0.919	(0.677, 1.248)	0.590	0.590
	Weighted median	0.932	(0.673, 1.292)	0.673	0.673
Deep vein thrombosis	IVW (multiplicative random effects)	1.164	(1.046, 1.296)	0.006	0.013
	Simple median	1.159	(1.009, 1.331)	0.037	0.086
	Weighted median	1.173	(1.014, 1.356)	0.032	0.112
Pulmonary embolism	IVW (multiplicative random effects)	1.346	(1.112, 1.630)	0.002	0.008
	Simple median	1.371	(1.098, 1.713)	0.005	0.038
	Weighted median	1.418	(1.135, 1.772)	0.002	0.015
Stroke	IVW (multiplicative random effects)	1.160	(1.082, 1.244)	3.05E-05	2.14E-04
	Simple median	1.156	(0.997, 1.341)	0.054	0.095
	Weighted median	1.149	(0.996, 1.325)	0.057	0.134

Table 9. Associations of genetic liability to cardiovascular diseases with cannabis use disorder.

Outcome	Exposures	SNP	IVW OR (95% CI)	P-value	MR PRESSO OR (95%CI)	P-value
Cannabis use disorders	Coronary artery disease	41	0.986 (0.911, 1.066)	0.715	NA[1]	NA
	Myocardial infarction	26	0.942 (0.853, 1.041)	0.243	0.924 (0.843, 1.012)[2]	0.089
	Atrial fibrillation	103	1.005 (0.959, 1.053)	0.846	NA[1]	NA
	Heart failure	11	0.996 (0.765, 1.297)	0.978	NA[1]	NA
	Deep venous thrombosis	10	1.015 (0.964, 1.069)	0.573	NA[1]	NA
	Pulmonary embolism	6	1.076 (0.967, 1.197)	0.181	NA[1]	NA
	Stroke	17	1.018 (0.832, 1.245)	0.862	NA[1]	NA

SNP, single nucleotide polymorphism; IVW, the inverse-variance weighted method; OR, odds ratio; CI, confidence interval; MR-PRESSO, MR-Pleiotropy Residual Sum and Outlier; NA, not available.

[1] No outlier detected.

[2] MR-PRESSO IV outliers detected: rs1870634.

Table 10. Mediation effect of cardiovascular confounders on cannabis use disorder-CVDs association.

Outcomes	Mediators	Total Effect	Mediation Effect		Mediated Proportion
		Effect Size (95%CI)	Effect Size (95%CI)	p-value	(%) (95% CI)
Coronary artery disease	Smoking	0.055 (0.008, 0.102)	0.018 (0.001, 0.035)	0.039	32.9 (1.7, 64.1)
	Alcohol		0.002 (-0.003, 0.008)	0.435	3.9 (-5.9, 13.6)
	BMI		0.015 (-0.004, 0.034)	0.114	27.8 (-6.7, 57.8)
	HDL		6.57E-03 (-0.004, 0.017)	0.203	11.9 (-6.4, 30.3)
	LDL		0.015 (0.003, 0.027)	0.012	27.9 (6.1, 49.7)
	TC		0.008 (-0.001, 0.018)	0.092	15.0 (-2.5, 32.5)
	TG		0.006 (-4.22E-04, 0.013)	0.066	11.5 (-0.8, 23.8)
	T2D		-0.007 (-0.019, 0.006)	0.293	-12.2 (-34.9, 10.5)
	Hypertension		0.017 (-0.003, 0.037)	0.091	30.8 (-4.9, 66.4)
	Depression		0.008 (-0.008, 0.024)	0.316	14.5 (-13.8, 42.8)
Myocardial infarction	Smoking	0.054 (0.014, 0.095)	0.025 (0.003, 0.046)	0.025	45.5 (5.8, 85.3)
	Alcohol		0.002 (-0.003, 0.008)	0.415	4.4 (-6.1, 4.8)
	BMI		0.014 (-0.003, 0.031)	0.115	25.7 (-6.3, 57.8)
	HDL		0.006 (-0.003, 0.015)	0.208	10.7 (-6.0, 27.4)
	LDL		0.015 (0.003, 0.027)	0.013	27.5 (5.8, 49.1)
	TC		0.008 (-0.001, 0.016)	0.095	13.9 (-2.4, 30.1)
	TG		0.007 (-3.68E-04, 0.014)	0.063	12.3 (-0.7, 25.2)
	T2D		-0.005 (-0.014, 0.005)	0.305	-9.2 (-26.7, 8.3)
	hypertension		0.013 (-0.003, 0.028)	0.104	23.5 (-4.8, 51.9)
	Depression		0.020 (0.004, 0.036)	0.014	36.8 (7.3, 66.4)
Atrial fibrillation	Smoking	0.06 (0.016, 0.105)	0.011 (-0.002, 0.025)	0.099	18.9 (-3.5, 41.3)
	Alcohol		0.002 (-0.002, 0.006)	0.263	3.7 (-2.8, 10.1)
	BMI		0.012 (-0.003, 0.027)	0.113	19.8 (-4.6, 44.2)
	HDL		3.59E-05 (-0.002, 0.002)	0.974	0.1 (-3.6, 3.7)
	LDL		3.25E-04 (-0.001, 0.002)	0.686	0.5 (-2.1, 3.1)
	TC		-1.55E-04 (-0.001, 0.001)	0.733	-0.3 (-1.7, 1.2)
	TG		-0.001 (-0.003, 0.003)	0.485	-1.4 (-5.3, 2.5)
	T2D		0.001 (-0.002, 0.004)	0.442	2.1 (-3.2, 7.4)
	hypertension		0.013 (-0.002, 0.028)	0.082	21.7 (-2.8, 46.1)
	Depression		0.004 (-0.006, 0.014)	0.417	6.6 (-9.3, 22.5)
Heart failure	Smoking	0.091 (0.042, 0.140)	0.020 (0.003, 0.038)	0.024	22.2 (2.9, 41.5)
	Alcohol		0.001 (-0.004, 0.007)	0.670	1.3 (-4.6, 7.2)
	BMI		0.018 (-0.004, 0.040)	0.111	19.5 (-4.5, 43.4)
	HDL		0.001 (-0.002, 0.003)	0.690	0.6 (-2.3, 3.5)
	LDL		0.005 (0.001, 0.010)	0.022	5.8 (0.8, 10.7)
	TC		0.003 (-0.001, 0.006)	0.105	3.1 (-0.6, 6.8)
	TG		0.004 (-2.89E-04, 0.008)	0.069	4.1 (-0.3, 8.4)
	T2D		-0.005 (-0.014, 0.005)	0.305	-5.5 (-15.9, 5.0)
	hypertension		0.019 (-0.002, 0.039)	0.075	27.4 (-2.7, 57.4)
	Depression		0.013 (0.001, 0.026)	0.035	14.7 (1.0, 28.3)

Deep venous thrombosis	Smoking	0.138 (0.029, 0.246)	0.016 (-0.008, 0.040)	0.196	11.5 (-5.9, 28.9)
	Alcohol		-0.014 (-0.031, 0.003)	0.103	-10.2 (-22.5, 2.1)
	BMI		0.015 (-0.004, 0.033)	0.120	10.6 (-2.8, 24.0)
	HDL		-0.001 (-0.006, 0.004)	0.678	-0.8 (-4.5, 2.9)
	LDL		1.44E-04 (-0.007, 0.007)	0.969	0.1 (-5.2, 5.4)
	TC		0.002 (-0.001, 0.004)	0.147	1.2 (-0.4, 2.8)
	TG		-0.003 (-0.009, 0.002)	0.195	-2.5 (-6.4, 1.3)
	T2D		-0.005 (-0.014, 0.005)	0.305	-3.6 (-10.5, 3.3)
	hypertension		0.008 (-0.003, 0.019)	0.148	5.9 (-2.1, 13.8)
	Depression		0.008 (-0.008, 0.024)	0.332	5.9 (-6.0, 17.1)
Pulmonary embolism	Smoking	0.313 (0.16, 0.465)	0.024 (-0.012, 0.060)	0.186	7.7 (-3.7, 19.1)
	Alcohol		-0.021 (-0.045, 0.003)	0.088	-6.6 (-14.2, 1.0)
	BMI		0.013 (-0.005, 0.030)	0.149	4.1 (-1.5, 9.6)
	HDL		-0.001 (-0.007, 0.005)	0.797	-0.3 (-2.2, 1.7)
	LDL		0.007 (-0.004, 0.018)	0.207	2.3 (-1.3, 55.8)
	TC		0.003 (-0.003, 0.009)	0.280	1.1 (-0.9, 3.0)
	TG		0.003 (-0.003, 0.009)	0.405	0.8 (-1.1, 2.7)
	T2D		-1.14E-04 (-0.005, 0.004)	0.961	0.0 (-1.5, 1.4)
	hypertension		0.005 (-0.006, 0.016)	0.364	1.6 (-1.9, 5.1)
	Depression		0.021 (-0.010, 0.052)	0.182	6.8 (-3.2, 16.7)
Stroke	Smoking	0.091 (0.031, 0.152)	0.016 (2.55E-04, 0.033)	0.046	17.9 (0.3, 35.6)
	Alcohol		0.005 (-0.001, 0.010)	0.082	5.3 (-0.7, 11.3)
	BMI		0.006 (-0.002, 0.014)	0.142	6.5 (-2.2, 15.2)
	HDL		0.002 (-0.001, 0.005)	0.258	2.2 (-1.6, 6.0)
	LDL		0.003 (-8.77E-05, 0.006)	0.057	3.3 (-0.1, 6.7)
	TC		0.002 (-0.001, 0.004)	0.147	1.8 (-0.6, 4.2)
	TG		9.40E-06 (-0.002, 0.002)	0.991	0.0 (-1.8, 1.8)
	T2D		-0.004 (-0.013, 0.004)	0.296	-4.9 (-14.0, 4.3)
	hypertension		0.025 (-0.002, 0.052)	0.075	27.4 (-2.7, 57.4)
	Depression		0.002 (-0.008, 0.013)	0.665	2.6 (-9.0, 14.1)

BMI, body mass index; HDL, high-density lipoprotein cholesterol; LDL, low-density lipoprotein cholesterol; TG, triglycerides; TC, total cholesterol.

R codes used in this study:

```
library(TwoSampleMR)

##select SNPs
exp_dat <- read.table(file = "cannabis use disorder.txt", header = T, na.strings = "NA")
exp_dat <- exp_dat[exp_dat$P < 5e-07,]
exp_dat$pval.exposure <- exp_dat$P
{exp_dat = clump_data(exp_dat,
                     clump_kb = 10000,
                     clump_r2 = 0.01,
                     clump_p1 = 1,
                     clump_p2 = 1,
                     pop = "EUR")}

write.table(exp_dat,file="cannabis use disorder.txt",quote=F,sep="\t",col.names=T, row.name
= F)

##unvariable MR
exposure_dat <-read_exposure_data(
  filename = 'SNP-cannabis-12.csv',
  sep = ',',
  snp_col = 'SNP',
  beta_col = 'beta',
  se_col = 'se',
  effect_allele_col = 'effect_allele',
  phenotype_col = 'Phenotype',
  units_col = 'units',
  other_allele_col = 'other_allele',
  eaf_col = 'eaf',
  samplesize_col = 'samplesize',
  ncase_col = 'ncase',
  ncontrol_col = 'ncontrol',
  gene_col = 'gene',
  pval_col = 'pval'
)

{ outcome_AF <- extract_outcome_data(
    snps = exposure_dat$SNP,
    outcomes = 'ebi-a-GCST006061')
  outcome_AF$outcome = "Atrial fibrillation"

  outcome_CAD <- extract_outcome_data(
    snps = exposure_dat$SNP,
    outcomes = 'ieu-a-7')
  outcome_CAD$outcome = "Coronary heart disease"

  outcome_MI <- extract_outcome_data(
    snps = exposure_dat$SNP,
    outcomes = 'ieu-a-798')
  outcome_MI$outcome = "Myocardial infarction"

  outcome_HF <- read_outcome_data(
    snps = exposure_dat$SNP,
    filename = "HERMES_Jan2019_HeartFailure_summary_data.txt",
```

```
    "\t",
    snp_col = "SNP",
    beta_col = "b",
    se_col = "se",
    effect_allele_col = "A1",
    other_allele_col = "A2",
    pval_col = "p",
    chr_col = "CHR",
    pos_col = "BP",
    eaf_col = "freq")
  outcome_HF$outcome = "Heart failure"

  outcome_Stroke <- extract_outcome_data(
    snps = exposure_dat$SNP,
    outcomes = 'ebi-a-GCST005838')
  outcome_Stroke$outcome = "Stroke"

  outcome_DVT <- extract_outcome_data(
    snps = exposure_dat$SNP,
    outcomes = 'ukb-b-12040')
  outcome_DVT$outcome = "Deep venous thrombosis"
  u_DVT = 9241/(9241+453692)
  outcome_DVT$beta.outcome <- outcome_DVT$beta.outcome/(u_DVT*(1-u_DVT))
  outcome_DVT$se.outcome <-outcome_DVT$se.outcome/(u_DVT*(1-u_DVT))

  outcome_PE <- extract_outcome_data(
    snps = exposure_dat$SNP,
    outcomes = 'ukb-b-16048')
  outcome_PE$outcome = "Pulmonary embolism"
  u_PE <- 3823/(3823+459110)
  outcome_PE$beta.outcome <- outcome_PE$beta.outcome/(u_PE*(1-u_PE))
  outcome_PE$se.outcome <- outcome_PE$se.outcome/(u_PE*(1-u_PE))
}

library(dplyr)
outcome_dat=dplyr::bind_rows(outcome_AF,outcome_CAD,outcome_DVT,outcome_HF,
                             outcome_MI,outcome_Stroke,outcome_PE)
dat <- harmonise_data(exposure_dat,outcome_CAD,action = 1)
mr_results <- mr(dat,method_list=c("mr_ivw_mre",
                                   "mr_ivw_fe",
                                   "mr_simple_median",
                                   "mr_weighted_median",
                                   "mr_egger_regression"
                                   ))
OR_mr_results=generate_odds_ratios(mr_results)

het=mr_heterogeneity(dat)
ple=mr_pleiotropy_test(dat)

library(ggplot2)
# Leave-one-out
res_loo <- mr_leaveoneout(dat,method = mr_ivw_mre)
OR_res_loo = generate_odds_ratios(res_loo)
p <- mr_leaveoneout_plot(res_loo)
ggsave(p[[1]], file="image/leave-PE.png", width=2.5, height=3)
```

```
##Scatter plot
res <- mr(dat, method_list=c("mr_ivw_mre","mr_simple_median","mr_weighted_median"))
p1 <- mr_scatter_plot(res, dat)
ggsave(p1[[1]], file="image/scatter-PE.png", width=8, height=4)

library(MRPRESSO)
mr_presso(BetaOutcome = "beta.outcome", BetaExposure = "beta.exposure",
        SdOutcome = "se.outcome", SdExposure = "se.exposure",
        OUTLIERtest = TRUE, DISTORTIONtest = TRUE, data = dat,
        NbDistribution = 1000,  SignifThreshold = 0.05)


#multivariable MR
library(MendelianRandomization)
MRInputObject <- mr_mvinput(bx = cbind(GX_exp,GX_adj),
                     bxse = cbind(Gx_SE_exp,Gx_SE_adj),
                     by = GY,
                     byse = GY_SE)

tem= mr_mvivw(MRInputObject, model = "random",
          correl = FALSE,distribution = "normal", alpha = 0.05)

tem
```

Effect of SNPs on each outcome.

SNP	EA	NEA	Exposure		CAD		MI		AF		HF		DVT		PE		Stroke	
			Beta	SE	Beta	SE	Beta	SE	Beta	SE	Beta	SE	Beta	SE	Beta	SE	Beta	SE
rs1392816	T	C	-0.0998	0.0184	-0.0101	0.010	-0.0158	0.0107	-0.0112	0.0075	-0.0023	0.0081	-0.0317	0.0154	-0.0221	0.0238	-0.0117	0.0085
rs1509378	A	G	0.0963	0.0189	0.0070	0.010	0.0065	0.0114	0.0057	0.0077	0.0229	0.0085	0.0088	0.0159	0.0604	0.0245	0.0097	0.0087
rs2624841	T	C	0.0897	0.0176	0.0152	0.011	0.0158	0.0120	0.0123	0.0079	0.0121	0.0082	0.0095	0.0157	0.0596	0.0243	0.0163	0.0089
rs72818514	T	C	-0.1828	0.0342	-0.0090	0.025	-0.0226	0.0269	0.0264	0.0177	-0.0046	0.0162	0.0234	0.0337	NA	NA	0.0013	0.0201
rs553920	T	C	0.1040	0.0198	-0.0017	0.010	4.05E-04	0.0113	0.0200	0.0082	0.0072	0.0092	-0.0279	0.0175	-0.0130	0.0271	-7.00E-04	0.0089
rs12536335	A	G	0.0916	0.0171	0.0164	0.009	0.0052	0.0105	0.0023	0.0073	0.0027	0.0079	0.0188	0.0151	0.0145	0.0234	0.0104	0.0083
rs55986679	A	T	0.1262	0.0216	0.0200	0.012	0.0167	0.0131	2.00E-04	0.0090	0.0106	0.0100	0.0443	0.0186	0.0692	0.0288	0.0060	0.0101
rs10886017	A	C	0.0990	0.0195	-0.0037	0.010	-0.0031	0.0116	0.0068	0.0081	0.0305	0.0091	0.0070	0.0173	0.0477	0.0268	9.00E-04	0.0087
rs3914059	T	C	-0.0850	0.0168	3.14E-04	0.009	0.0015	0.0104	-0.0113	0.0071	-0.0023	0.0079	-0.0068	0.0152	-0.0033	0.0235	-0.0162	0.0078
rs9787909	A	C	0.1137	0.0225	-0.0070	0.012	0.0012	0.0129	0.0050	0.0092	0.0114	0.0102	0.0497	0.0192	0.0043	0.0296	0.0216	0.0098
rs17271123	T	G	0.1284	0.0252	0.0011	0.011	4.32E-05	0.0119	7.00E-04	0.0081	0.0067	0.0085	0.0197	0.0161	0.0341	0.0248	0.0291	0.0099
rs590076	A	G	0.0874	0.0171	0.0062	0.010	0.0017	0.0105	0.0076	0.0075	0.0053	0.0083	0.0045	0.0157	0.0462	0.0242	-0.0125	0.0084

EA, effect allele; NEA, non-effect allele; CAD, Coronary artery disease; MI, myocardial infarction; AF, atrial fibrillation; HF, heart failure; DVT, deep vein thrombosis; PE, pulmonary embolism.
