# Supplementary figures and images for: Association of cannabis use disorder with cardiovascular diseases: A two-sample Mendelian randomization study
Source: Front Cardiovasc Med. 2022 Oct 6;9:966707. doi: 10.3389/fcvm.2022.966707 (PMC9582269; doi:10.3389/fcvm.2022.966707)

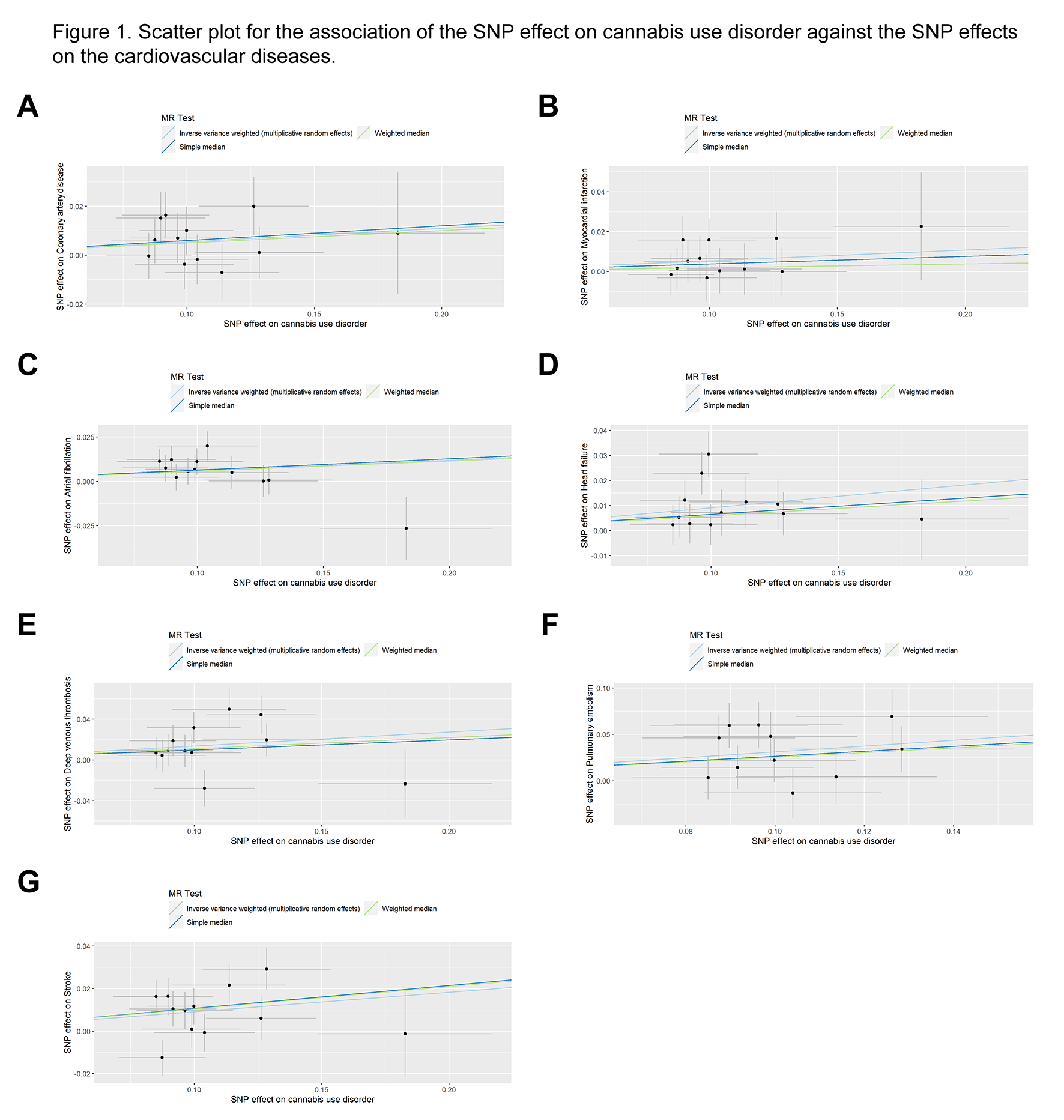

Supplement: Supplementary file 2 [file Image_1.TIF]

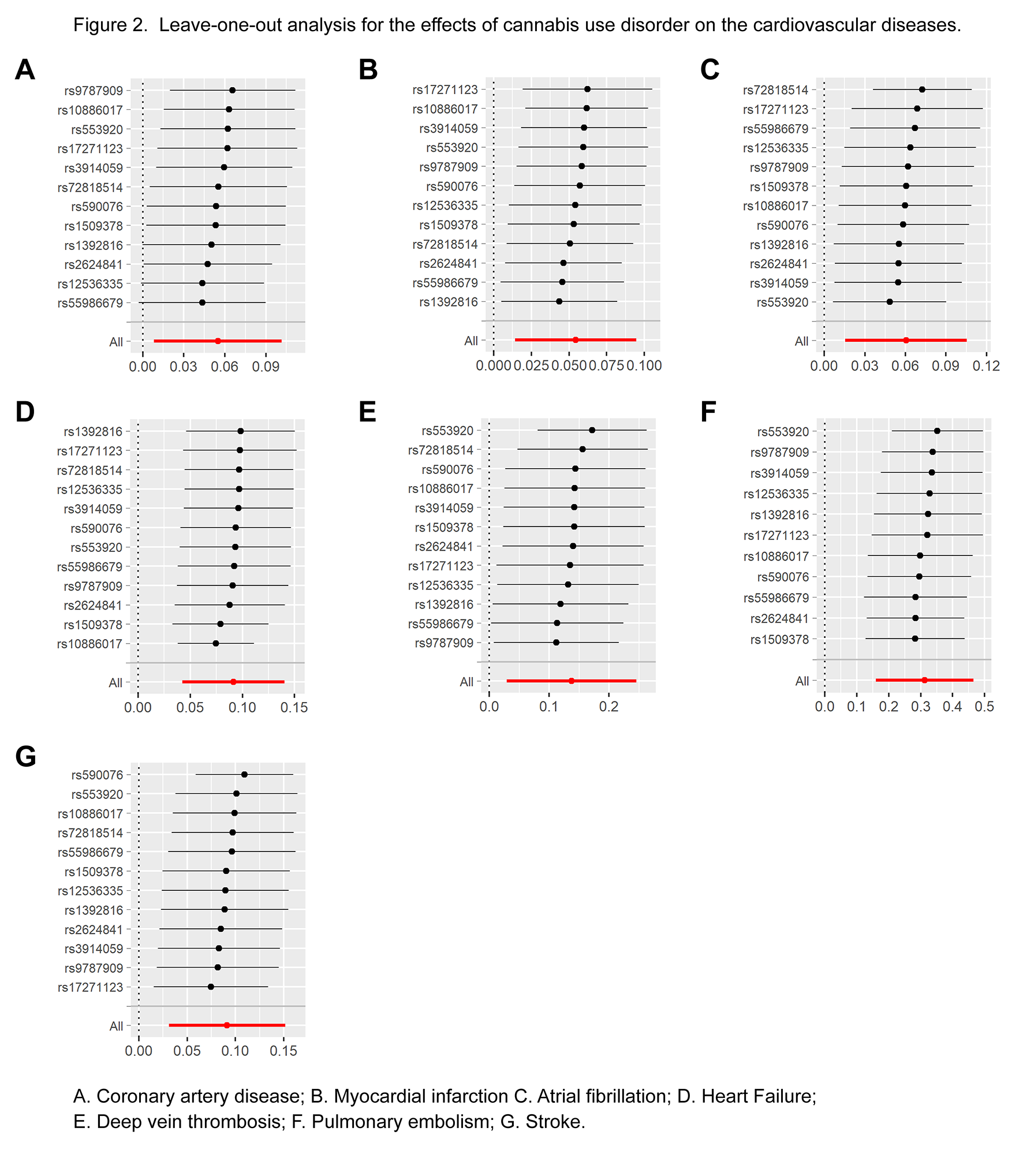

Supplement: Supplementary file 3 [file Image_2.TIF]
